# Air pollution, aeroallergens and admissions to pediatric emergency room for respiratory reasons in Turin, northwestern Italy

**DOI:** 10.1186/s12889-016-3376-3

**Published:** 2016-08-05

**Authors:** Roberto Bono, Valeria Romanazzi, Valeria Bellisario, Roberta Tassinari, Giulia Trucco, Antonio Urbino, Claudio Cassardo, Consolata Siniscalco, Pierpaolo Marchetti, Alessandro Marcon

**Affiliations:** 1 Department of Public Health and Pediatrics, University of Turin, via Santena, 5 bis, 10126 Turin, Italy; 2 Pediatrics Emergency, Regina Margherita Children’s Hospital, Piazza Polonia, 94, 10126 Turin, Italy; 3 Department of Physics, University of Turin, Via P. Giuria, 1, 10125 Turin, Italy; 4 Department of Life Sciences and Systems Biology, University of Turin, Viale P. A. Mattioli, 25, 10125 Turin, Italy; 5 Unit of Epidemiology and Medical Statistics, Department of Diagnostics and Public Health, University of Verona, Strada le Grazie, 8, Verona, Italy

**Keywords:** Airborne pollutants, Pollens, Time-series analysis, Pediatric emergency room, Short-term respiratory effects

## Abstract

**Background:**

Air pollution can cause respiratory symptoms or exacerbate pre-existing respiratory diseases, especially in children. This study looked at the short-term association of air pollution concentrations with Emergency Room (ER) admissions for respiratory reasons in pediatric age (0–18 years).

**Methods:**

Daily number of ER admissions in a children’s Hospital, concentrations of urban-background PM_2.5_, NO_2_, O_3_ and total aeroallergens (Corylaceae, Cupressaceae, Gramineae, Urticaceae, Ambrosia, Betula) were collected in Turin, northwestern Italy, for the period 1/08/2008 to 31/12/2010 (883 days). The associations between exposures and ER admissions were estimated, at time lags between 0 and 5 days, using generalized linear Poisson regression models, adjusted for non-meteorological potential confounders.

**Results:**

In the study period, 21,793 ER admissions were observed, mainly (81 %) for upper respiratory tract infections. Median air pollution concentrations were 22.0, 42.5, 34.1 μg/m^3^ for urban-background PM_2.5_, NO_2_, and O_3_, respectively, and 2.9 grains/m^3^ for aeroallergens. We found that ER admissions increased by 1.3 % (95 % CI: 0.3-2.2 %) five days after a 10 μg/m^3^ increase in NO_2_, and by 0.7 % (95 % CI: 0.1-1.2 %) one day after a 10 grains/m^3^ increase in aeroallergens, while they were not associated with PM_2.5_ concentrations. ER admissions were negatively associated with O_3_ and aeroallergen concentrations at some time lags, but these association shifted to the null when meteorological confounders were adjusted for in the models.

**Conclusions:**

Overall, these findings confirm adverse short-term health effects of air pollution on the risk of ER admission in children and encourage a careful management of the urban environment to health protection.

**Electronic supplementary material:**

The online version of this article (doi:10.1186/s12889-016-3376-3) contains supplementary material, which is available to authorized users.

## Background

Over the last decades, the prevalence of respiratory diseases, and in particular of asthma and allergies, has increased considerably, especially in industrialized countries [1, 2]. The etiology of respiratory diseases is multifactorial and includes, among others, interactions between genetic predisposition and environmental factors [3]. The environmental dynamics, characterized by climate change, qualitative and quantitative aspects of chemical air pollution and airborne pollens, may partially explain the increased incidence of respiratory symptoms and respiratory diseases during the last years [4]. The short-term respiratory effects of air pollution include decreases in pulmonary function [5], increases in inflammatory biomarkers [6] and respiratory symptoms [7, 8], exacerbations of chronic obstructive pulmonary disease (COPD), infections [9, 10], school absenteeism [11] and respiratory mortality [12, 13].

The respiratory system is a primary target of air pollution. In children, the small airway caliber allows for a higher chance of being affected by inflammation resulting from air pollution [14, 15]. Due to their respiratory rates, children breathe a proportionately greater volume of air than adults and their oxygen demand is significantly higher, as well as their respiration rates. Young people also spend more time engaged in intense activities than adults, often outdoors and during midday when air pollution levels tend to be higher. As a result, children inhale more pollutants per kilogram of body weight. Irritation caused by air pollutants that would produce only a slight response in an adult can result in potentially significant obstruction in the airways of a young child [16].

The environmental risk factors that may have an impact on children’s respiratory health, especially in urban areas, include chemical outdoor pollution, aeroallergens, indoor air pollution including environmental tobacco smoke, microorganisms such as virus and bacteria that infect the airways. The latter can exacerbate or re-exacerbate their manifestations in presence of other risk factors.

Several epidemiological studies have documented a positive association between exposure to particulate air pollution and respiratory symptoms of cough and wheeze, especially among children [17, 18]. In this regard, the findings from two Swiss studies showed that the reduction of exposure to particulate matter (PM) <10 μm in aerodynamic diameter (PM_10_) contributes to improved respiratory health, observed through fewer cases of chronic cough in children [19] and through fewer cough, wheezing and breathlessness in adults [20]. Exposure to ozone (O_3_) at environmental concentrations is associated with lung function decrease and respiratory symptoms including cough, shortness of breath and pain on deep inspiration [21]. Nitrogen dioxide (NO_2_) concentrations have also been associated with cough, wheeze and breath shortness in children. Residential traffic-related air pollution exposure is associated with reduced expiratory flows in schoolchildren [7, 22]. Variations in lung function that mirror changes in PM exposure have been reported in children who move to areas with different air pollution levels [23].

Pollen is a well know trigger of allergies and asthma aggravation, and actually has a changing profile; in fact new pollen types have emerged following the cultivation and spread of exotic ornamental plants in public and private places [24]. Moreover, global climate change has been linked to an earlier onset and an extended duration of the pollens season, to an increase in pollen production, and a stronger allergenicity for some pollen types [25]. Thunderstorm asthma epidemics may be triggered by pollen grains rupture in the atmosphere and the entrapment of respirable-size particles in the outflows of air masses at ground level [24, 25]. Increasing pollution is responsible for an increase in pollen-induced respiratory allergy, including asthma, because of airway inflammatory reaction and the passage of pollen grains into the lower respiratory tract [24].

The aim of this study was to analyze the short-term relationships between hospital emergency room (ER) admissions for respiratory diseases in children and concentrations of NO_2_, PM_2.5_, O_3_, and aeroallergens, in Turin, Italy, between 2008 and 2010.

## Methods

Turin, the capital of Piedmont region (North-Western Italy) has 900,000 inhabitants, is located at 200 m above sea level and it is one of the most polluted Italian cities [26–29] Additional file 1: Figure S1. Daily data for the period 01/08/2008 to 31/12/2010 (883 days) for the city of Turin were collected or derived as described below. The locations of the data sources are shown in Additional file 1: Figure S1.

### ER admissions for respiratory diseases

Daily data on ER admissions (date of admission, primary diagnosis and diagnostic code) for 19 respiratory diseases to “Regina Margherita” Pediatric Hospital of Turin were collected (age range 0–18 years). Diagnoses were coded according to the International Classification of Disease (ICD) 9th edition (Table 1).

**Table 1:** Distribution of daily ER admissions, for the diagnoses of the respiratory diseases considered in the analysis, during the study period (883 days)

Respiratory disease diagnoses			total n. of admissions	daily n. of admissions		
Group	Description	ICD-IX-CM code	count	mean ± SD	median	min-max
Upper respiratory tract infections	Acute rhino pharyngitis	460	17684	18.1 ± 9.0	17	0-45
	Acute pharyngitis	462				
	Acute tonsillitis	463				
	Acute laryngitis without obstruction	46400				
	Acute laryngitis with obstruction	46401				
	Acute upper respiratory infections	4658				
Lower respiratory tract infections	Acute bronchitis	4660	1989	3.0 ± 3.2	2	0-20
	Acute bronchiolitis other infectious agents	46619				
	Flu with respiratory manifestations	4871				
	Bronchitis	490				
Deep lung infections	Viral pneumonia	4809	839	2.1 ± 2.1	2	0-8
	Bacterial pneumonia	4829				
	Bronchopneumonia	485				
	Pneumonia	486				
Asthma	Asthma, without status asthmatics	49390	1281	1.5 ± 1.6	1	0-7
	Asthma, with status asthmatics	49391				
Total			21793	24.7 ± 11.7	23	0-80

International classification of diseases, 9th edition, clinical modification (ICD-IX-CM) codes

### Meteorological data

Meteorological data derived from the station placed on the roof of the Department of Physics of the University of Turin, located at about 1 km from the city center. The station is permanently active since 1989 in order to collect and display in real time the weather data in the urban surface layer of the city. The station is equipped with the instruments reported in Additional file 1: Table S1. Data are collected every 5 s by the acquisition system, and subsequently averaged every 5 min and stored in an electronic archive. Data acquired in this way were aggregated in a daily form for the subsequent analysis.

### Chemical air pollution data

Daily concentrations of NO_2_, PM_2.5_ and O_3_ were derived from hourly data collected at the urban background monitoring station “Lingotto” located in Turin (viale Augusto Monti, 21) by the Local Environmental Protection Agency (ARPA Piemonte), coordinated by the regional air pollution service of Piedmont Region, according to the current European legislation (DIR 2008/50/ECX).

### Aeroallergen data

Among the pollen taxa usually considered in aerobiological monitoring for being allergenic, Corylaceae, Cupressaceae, Gramineae, Urticaceae, Ambrosia, and Betula were quantified in this study. Daily data were derived from a station located 12 m above the ground, as required by the standard [30], on the flat roof of a building located in a semi-central area of the city of Turin. In this site, atmospheric circulation is local and not affected by surrounding obstacles such as walls or other types of protection. The station is equipped with a HIRST sampler, which consists of three main parts: a swivel head, a suction pump and a deposition drum (the sampling part), which rotates at 2 mm / h with 7 days of power reserve. Weekly, a specific adhesive tape is fixed on the drum. This tape captures the aeroallergens avoiding any loss for rebound or natural detachment. The air pump provides a constant airflow of 10 L / min inside the sampler, equivalent to 14.4 m^3^ each 24 h. Daily aeroallergen counts were carried out at the Department of Life Science and System Biology, University of Turin, and expressed as concentrations (grains/m^3^). For the statistical analysis, daily total aeroallergen concentrations were obtained as the sum of the concentrations of the single aeroallergen types.

### Statistical Analysis

Quantitative variables were summarized with means ± SD, medians with interquartile ranges (IQR), and minimum and maximum values. The interquartile ratio (IQR/median ratio) was also computed in order to compare variability across different air pollutants. Linear correlations among exposure variables were evaluated using Pearson’s r coefficients.

The association between daily ER admission counts for all diagnoses in Table 1 combined (dependent variable) and air pollution exposure variables were analyzed using Generalized Linear Models (GLMs) fitting a non-stationary Poisson process [30, 31]. We used the following model:

$$ \log \left({\lambda}_t\right)=\alpha +{\displaystyle \sum_{i=1}^k}\beta {X}_i+NS(Z) $$

Where *λ*_*t*_ denotes the count of daily ER admissions at day *t*, *α* is a constant, *β* is the vector of estimated parameters, *X*_*i*_ is the matrix of *k* independent variables (exposure and adjustment variables), and *NS(Z*_*t*_*)* is a natural spline smoothing function of calendar day *Z* with 14° of freedom (df), [31] which was included to take the medium/long term trend into account [30, 31]. The number of df of the smoothing function was chosen by minimizing the sum of the absolute values of the partial autocorrelation function (PACF) of the residuals [11, 30, 31]. Day of the week was included when estimating the smoothing function to remove the 7-day positive correlation across PACF residuals. To avoid overfitting, the maximum number of df allowed was 15, which corresponds to about 6 df per calendar year (60-day windows) [32].

The adjustment variables considered were: a) day of the week, b) influenza outbreaks, defined as days when influenza incidence was greater than 2‰ [33], which were computed by the Regional Reference Service of Epidemiology for the Surveillance, Prevention and Control of Infectious Diseases, ASL Alessandria, Italy Reference Service Regional Epidemiology and Infectious Disease (SeREMI), c) holidays (4-level variable coded as: Christmas and Easter; 3 days around Christmas and Easter; other holidays; other days), d) summer population decrease (from Saturday before Mid-August to the next Sunday for a total of 16 days/year; from 16 July to 31 August, except for the aforementioned period; all other days) [34], e) average daily temperature, f) average relative humidity, and g) cumulative daily precipitations. The following models were fit to the data:

A) One exposure variable + medium/long trend function + non meteorological variables (day of the week, influenza outbreaks, holidays and summer population decrease) (*single-pollutant models*);
B) Model A + meteorological variables (daily temperature, daily relative humidity, cumulative daily precipitations). Temperature and relative humidity were modeled using natural splines with 3 and 2° of freedom, respectively. The number of df was chosen using the PACF criterion as above. Daily precipitations were binary coded (present if ≥1 mm; absent otherwise);
C) One chemical pollutant (PM_2.5_, NO_2_ or O_3_) + aeroallergens + medium/long trend function + non meteorological variables (*two-pollutant models)*. To avoid multicollinearity, two-pollutant models only combined one chemical pollutant per time and aeroallergens, because correlations across chemical pollutants were very strong (Pearson’s coefficients of correlation in absolute value |r| >0.50).

Exposure variables were included in the models at single time lags, from the same day when ER admissions were evaluated (Lag 0) to 5 days before (Lag 5). Associations between exposure variables (10 μg/m^3^ increase in PM_2.5_, NO_2_, O_3_ concentrations; 10 grains/m^3^ increase in aeroallergen concentrations) and ER admissions were reported with rate ratios (RR) with 95 % confidence intervals (CI).

## Results

**Fig. 1:**
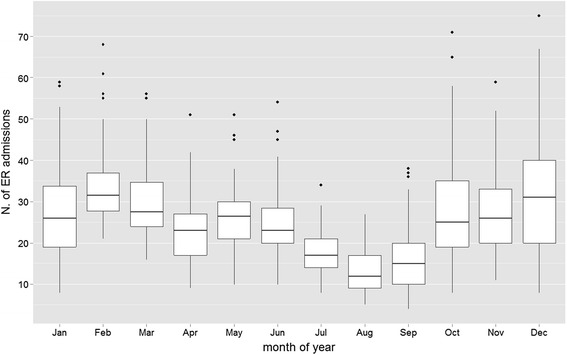
Monthly distribution of daily ER admissions for respiratory diseases during the study period

In the study period, 21,793 pediatric ER admissions for respiratory diseases were observed, mainly (81 %) for infections of the upper airways (Table 1). Figure 1 shows average daily ER admissions by month of the year. The cold months showed the highest frequency of ER admissions, probably due to the more frequent outbreaks of colds. Table 2 shows a general description of the daily concentrations of airborne pollutants during the study period. NO_2_ concentration showed the highest mean absolute levels and, overall, the air pollution concentrations observed underline the poor air condition in Turin as compared to the rest of Europe [26, 35].

**Table 2:** Distribution of daily concentrations of air pollution and aeroallergens at the Lingotto urban background monitoring station during the study period (883 days)

Exposure variable	available data (days)	median (IQR)	interquartile ratio	min-max	mean ± SD	EU annual reference value^b^
PM_2.5_ (μg/m^3^)	833	22.0 (30.0)	1.4	4–157	32.0 ± 26.2	25
NO_2_ (μg/m^3^)	851	42.5 (32.0)	0.8	7.4–192.9	48.3 ± 25.0	40
O_3_ (μg/m^3^)	858	34.1 (53.6)	1.6	1.8–123.3	9.6 ± 29.1	
aeroallergens (grains/m^3^)^a^	826	2.9 (19.6)	6.7	0–271.9	16.1 ± 29.6	

^a^includes Corylaceae, Cupressaceae, Gramineae, Urticaceae, Ambrosia, Betula

^b^European Union (EU) Directive 2008/50/CE10 (http://ec.europa.eu/environment/air/quality/legislation/directive.htm)

**Fig. 2:**
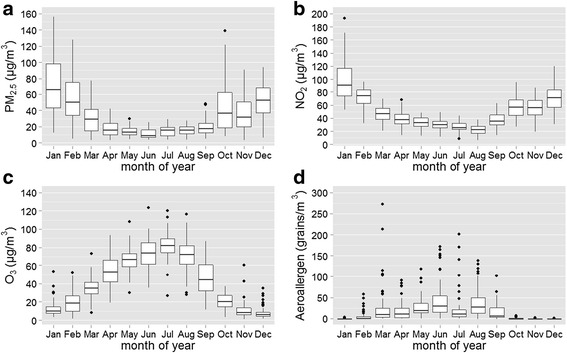
Monthly distribution of daily PM_2.5_, NO_2_, O_3_ and aeroallergen concentrations (panels **a** − **d**, respectively) during the study period

Aeroallergen concentrations showed larger variability than chemical air pollution concentrations: the interquartile ratio for aeroallergens was 4 (O_3_) to 8-fold (NO_2_) the interquartile ratio of the chemical pollutants. Both PM_2.5_ and NO_2_ (Fig. 2a and b) showed a prevailing maximum level during the coldest months, which is a typical behavior of primary pollutants. An opposite trend was shown by O_3_, with higher concentrations in summertime (Fig. 2c). The concentrations of aeroallergens were high in the warm season, and virtually absent in winter (Fig. 2d).

**Fig. 3:**
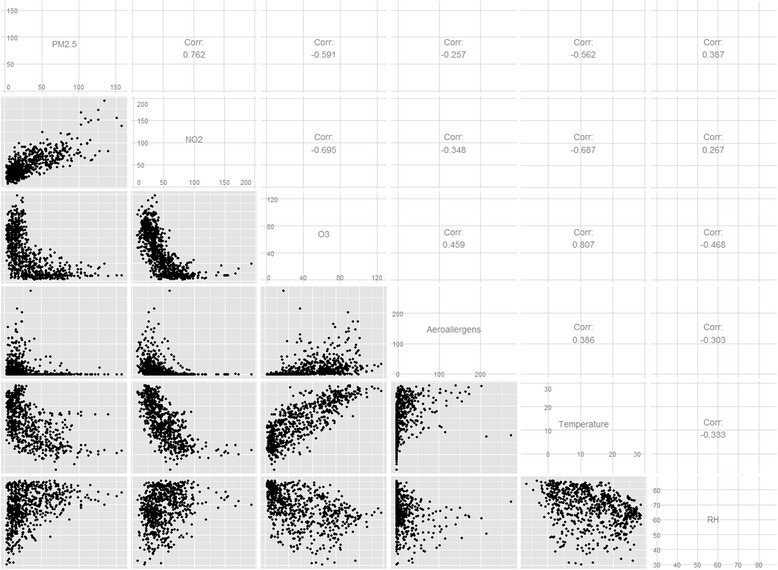
Pairwise distributions and Pearson’s r correlation coefficients of air pollutant concentrations and meteorological variables. All the hypothesis tests of no correlation were statistically significant (all *p* <0.001)

There was a strong positive linear correlation between PM_2.5_ and NO_2_ (*r* = 0.762, *p* < 0.001). The negative correlations of O_3_ with PM_2.5_ and NO_2_ were also strong (*r* = −0.591 and −0.695, respectively, all *p* < 0.001) (Fig. 3). The correlations between chemical pollutants and aeroallergens were weaker (|r| between 0.257 and 0.459).

Table 3 shows the mean number of daily ER admissions, air pollution and aeroallergen concentrations according to the potential confounders considered. ER admissions were more frequent during weekends, holidays, and during influenza outbreaks than during the other days. They were also more likely in days with lower temperatures and more extreme (low and high) relative humidity levels. PM_2.5_ and NO_2_ were positively associated with temperature, whereas the opposite was true for O_3_ and aeroallergens. As expected, airborne pollution was lower during rainy days.

**Table 3:** Distribution of the daily number of ER admissions for respiratory diseases and daily concentrations of chemical air pollution and aeroallergens during the study period, according to the potential confounders considered in the analysis^a^

	ER admissions (counts)	PM_2.5_ (μg/m^3^)	NO_2_ (μg/m^3^)	O_3_ (μg/m^3^)	Aeroallergens (grains/m^3^)
Day of the week					
Monday	22.7 ± 10.2	30.4 ± 26.5	47.2 ± 25.1	38.2 ± 27.7	12.7 ± 20.9
Tuesday	21.4 ± 9.8	30.8 ± 25.4	50.3 ± 24.5	37.8 ± 28.6	14.9 ± 28.8
Wednesday	20.4 ± 9.6	32.5 ± 25.4	51.0 ± 23.8	38.9 ± 29.2	18.8 ± 38.1
Thursday	21.6 ± 9.7	35.1 ± 27.8	52.9 ± 25.9	38.9 ± 30.2	15.1 ± 23.0
Friday	23.4 ± 11.1	33.3 ± 25.0	50.9 ± 24.7	39.9 ± 29.7	17.2 ± 32.1
Saturday	31.1 ± 12.4	31.5 ± 26.0	45.0 ± 25.1	42.2 ± 29.8	18.2 ± 33.4
Sunday	32.2 ± 12.8	30.4 ± 27.4	40.9 ± 24.0	41.3 ± 28.5	15.8 ± 27.4
*p*	<0.001	0.79	0.002	0.87	0.73
Holidays					
Christmas and Easter	47.6 ± 17.5	25.4 ± 15.2	45.1 ± 27.5	32.0 ± 29.9	1.0 ± 1.9
3 days before/after Christmas and Easter	34.9 ± 16.3	37.9 ± 21.7	58.6 ± 25.8	25.7 ± 23.7	6.4 ± 15.8
Other holidays	35.6 ± 15.6	37.4 ± 27.7	49.3 ± 22.7	32.2 ± 30.4	12.6 ± 1.9
Other days	23.8 ± 10.9	31.6 ± 26.4	48.0 ± 25.0	40.4 ± 29.1	16.6 ± 30.2
* p*	<0.001	0.39	0.23	0.02	0.20
Influenza outbreaks					
No	19.9 ± 9.0	22.8 ± 21.0	36.8 ± 16.9	55.2 ± 27.4	23.8 ± 31.9
Yes	31.0 ± 12.0	43.9 ± 27.5	63.0 ± 25.8	19.9 ± 16.4	6.3 ± 23.0
* p*	<0.001	<0.001	<0.001	<0.001	<0.001
Summer population decrease					
2 weeks around 15 August	13.4 ± 4.71	15.8 ± 5.6	20.7 ± 6.3	66.1 ± 15.4	34.7 ± 28.5
From 16/7 to 31/8 (except 2 weeks around 15 August)	14.4 ± 5.8	14.5 ± 6.4	26.1 ± 8.5	78.0 ± 19.3	29.1 ± 31.6
Other days	26.4 ± 11.6	34.5 ± 27.1	52.5 ± 24.5	33.7 ± 26.3	13.4 ± 28.7
* p*	<0.001	<0.001	<0.001	<0.001	<0.001
Temperature^b^ (°C)					
−6.4, 6.4	29.1 ± 11.6	57.3 ± 28.3	78.9 ± 27.6	12.1 ± 8.8	0.8 ± 2.5
6.5, 12.8	28.1 ± 11.3	31.7 ± 21.5	51.7 ± 15.2	23.3 ± 17.3	10.1 ± 31.9
12.9, 20.3	24.8 ± 11.5	27.5 ± 23.9	43.4 ± 17.1	41.2 ± 20.2	14.9 ± 21.6
20.4, 28.5	19.5 ± 8.2	14.9 ± 6.8	29.4 ± 9.5	73.7 ± 18.4	32.5 ± 39.7
* p*	<0.001	<0.001	<0.001	<0.001	<0.001
Cumulative precipitation					
<1 mm	25.4 ± 11.4	35.5 ± 28.0	51.5 ± 26.2	38.0 ± 33.7	16.0 ± 29.9
≥1 mm	25.2 ± 11.5	25.5 ± 19.2	45.0 ± 20.1	33.7 ± 26.0	9.5 ± 27.3
* p*	0.87	<0.001	0.01	0.10	0.02
Relative humidity ^b^(%)					
30.4, 60.4	26.4 ± 10.3	17.8 ± 13.1	41.6 ± 17.9	52.1 ± 24.6	25.1 ± 34.3
60.5, 68.6	23.1 ± 11.6	25.5 ± 18.9	43.2 ± 22.2	50.9 ± 31.3	21.8 ± 37.5
68.7, 76.4	25.3 ± 11.3	44.7 ± 30.5	56.9 ± 32.1	28.3 ± 23.0	9.6 ± 25.4
76.5, 85.5	26.7 ± 12.1	42.5 ± 28.8	58.4 ± 22.1	16.7 ± 15.9	1.9 ± 6.0
* p*	0.01	<0.001	<0.001	<0.001	<0.001

^a^mean ± SD reported; overall *p*-values were calculated using non-parametric Kruskall-Wallis tests, under the null hypothesis that the distribution of a variables is homogeneous among the strata of a potential confounder

^b^ coded in groups according to the quartiles of their frequency distribution

**Fig. 4:**
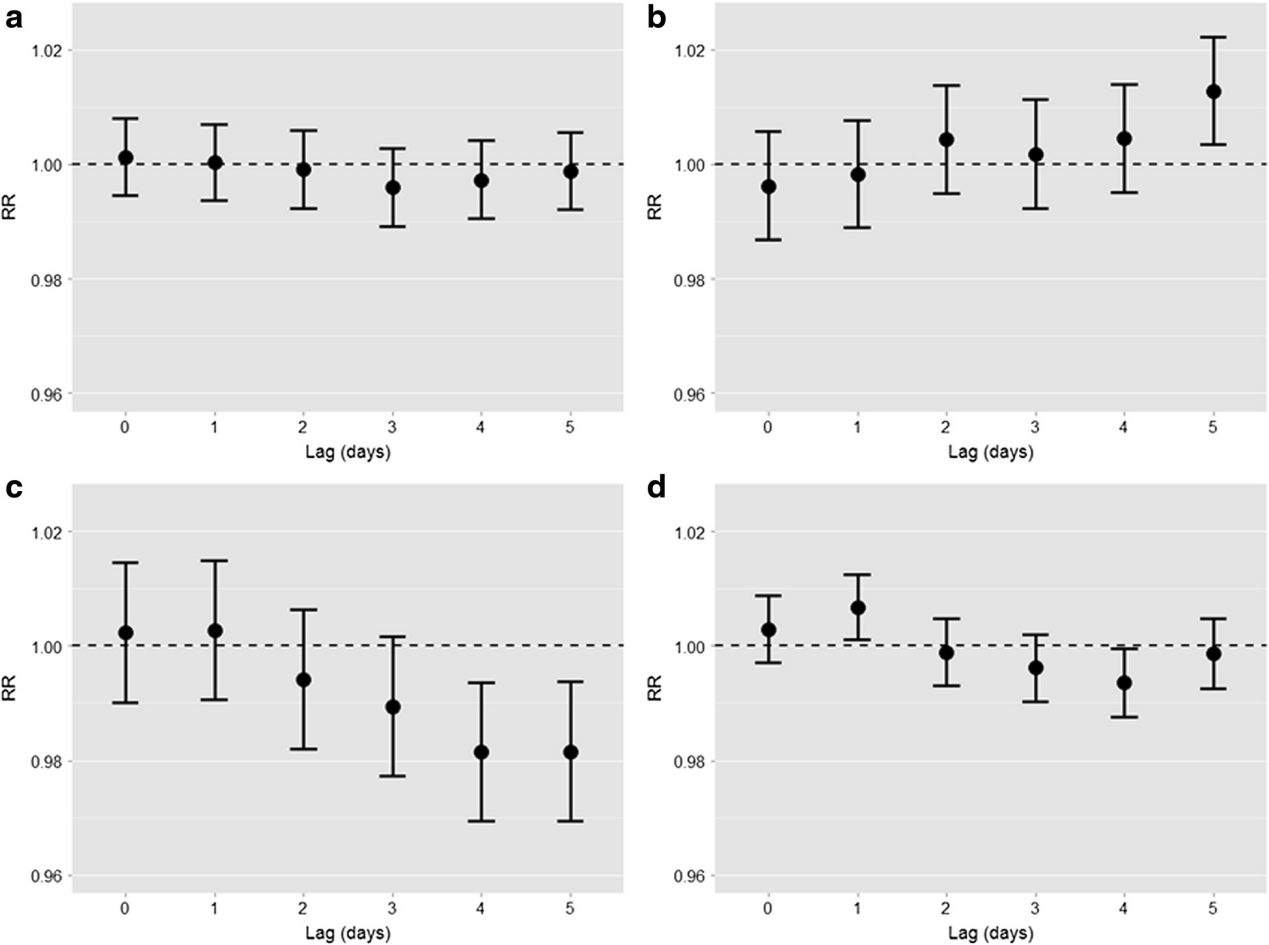
Estimates of association of daily PM_2,5_, NO_2_, O_3_, and aeroallergen concentrations (panels **a** − **d**, respectively) with ER admissions for respiratory diseases at different time lags, adjusted for medium/long-term trend function, day of the week, influenza outbreaks, holidays and summer population decrease.* *Single-pollutant models, see “model A” in the Statistical analysis section. Relative risks (RR) with 95%CIs are given for a 10 μg/m^3^ increase in PM_2,5_, NO_2_, O_3_ concentrations or a 10 grains/m^3^ increase in aeroallergen concentrations

**Fig. 5:**
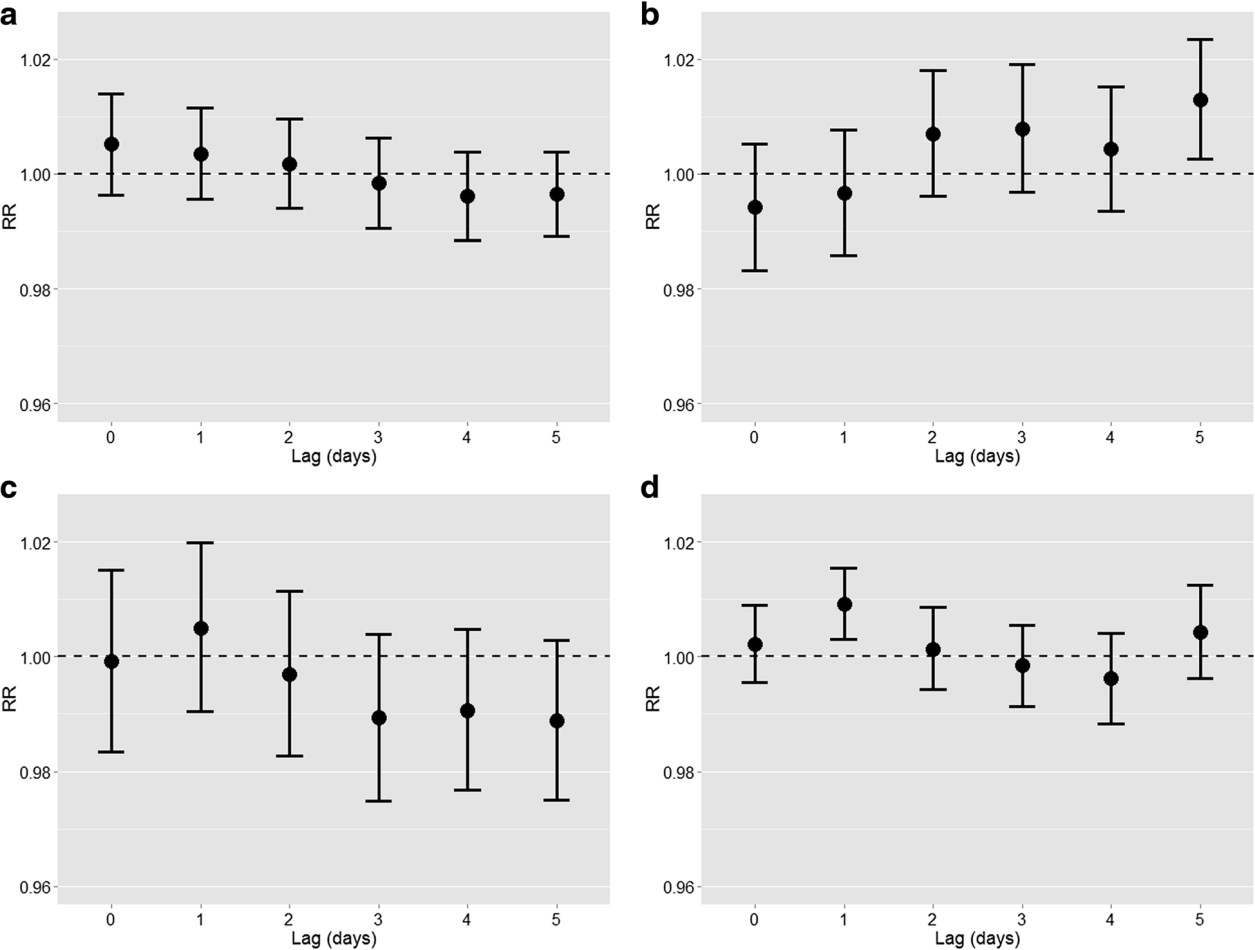
Estimates of association of daily PM_2,5_, NO_2_, O_3_, and aeroallergen concentrations (panels **a** − **d**, respectively) with ER admissions for respiratory diseases at different time lags, adjusted for medium/long-term trend function, day of the week, influenza outbreaks, holidays, summer population decrease, and for the meteorological variables (daily temperature, daily relative humidity, cumulative daily precipitations).* * Single-pollutant models, see “model B” in the Statistical analysis section of the article. Relative risks (RR) with 95%CIs are given for a 10 μg/m^3^ increase in PM_2,5_, NO_2_, O_3_ concentrations or a 10 grains/m^3^ increase in aeroallergen concentrations

The associations between exposure variables and ER admissions for respiratory diseases, adjusted for non-meteorological potential confounders (model A), is described in Fig. 4. There was no statistically significant association of PM_2.5_ and ER admissions at any time lag (Fig. 4a). Instead, an increase of 10 μg/m^3^ of NO_2_ concentrations (Fig. 4b) was associated with a significant 1.3 % (95 % CI: 0.3-2.2 %) increase of ER admissions after 5 days (lag 5). O_3_ concentrations were significantly negatively associated with ER admissions for respiratory diseases, starting from lag 4 (Fig. 4c). Finally, a 0.7 % (95 % CI: 0.1–1.2 %) increase of ER admissions was observed 1 day after (lag 1) an increase of 10 grains/m^3^ of aeroallergens (Fig. 4d). When meteorological variables were also included as adjustment variables in the analyses, the results were consistent, with the exception that the negative associations between O_3_ (lags 4–5) and aeroallergen (lag 4) concentrations and ER admissions shifted to the null (Fig. 5). Joint models including individual chemical air pollutants and aeroallergens confirmed the main models results completely (Additional file 1: Figure S2), suggesting that the observed associations of chemical pollutants and aeroallergens with ER admissions were independent.

## Discussion and conclusion

The main purpose of this study was to analyze in the selected period the trend of ER admissions for respiratory reasons in a Children’s Hospital in Turin and the relationships of ER admissions with urban background chemical air pollutants and aeroallergens.

To achieve this objective, we considered the monitoring site of chemical air pollution where the data were the most complete for the study period and which was the closest to the pediatric emergency room (Additional file 1: Figure S1).

Among the 19 diseases diagnosed at the moment of the access to pediatric emergency room, as expected the upper respiratory tract infections was the most frequent reason for access to the emergency room. These infectious diseases of viral etiology are very common in pediatric age also because children generally attend kindergarten, nurseries and schools and they are therefore more exposed both to the etiological agents and to environmental risk factors such as poor air quality. Air pollution in fact, as well as tobacco smoke, can inhibit defensive mechanisms against oxidative stress [7, 15] and inflammation of the upper respiratory tract, which can favor the development of respiratory virus infections.

Despite the fact that the concentration of PM_2.5_ appears quite high when compared to European levels [29], this pollutant did not reveal any significant short-term association with ER pediatric admission, as documented by other authors [36]. Instead, NO_2_ showed a positive significant association with ER admissions, but only after 5 days (lag 5). Also Li et al. have shown a positive association between NO_2_ air pollution at lag 5 and ER admission in children of Detroit (MI, U.S.A.) in 2011 [37]. However, in contrast to what we observed, they have also shown a positive and significant association between PM_2.5_ concentrations and ER admissions. This does not seem to depend primarily on the average concentrations of PM_2.5,_ which were much lower than in our study area, and it may be due to a different composition of particulate, perhaps more toxic in the City of Detroit than in Turin.

The adjusted estimates of relative risk for the effect of O_3_ were significantly less than one, seemingly suggesting a little protective effect. In 2009, Jerrett et al. also showed how relative risk for the effect of ozone on the risk of death from cardiovascular causes were significantly less than 1.0 [38].

Such beneficial influence of ozone, however, is currently completely to exclude from toxicological point of view. In experimental studies, O_3_ can increase airway inflammation [39] and can worsen pulmonary function and gas exchange [40]. In addition, exposure to elevated concentrations of tropospheric O_3_ has been associated with numerous adverse health effects, including the induction [26] and exacerbation [27, 28] of asthma, pulmonary dysfunction [33, 34] and hospitalization for respiratory reasons [31]. In our study, the apparent protective effect of O_3_ seems to be due to a confounding by meteorology, or to the fact that O_3_ acts as a mediator of the effect of temperature. In fact, when temperature, relative humidity and precipitations were included in the models as adjustment factors, the associations between O_3_ and ER admissions shifted to the null. Measurements of PM_2.5_ and NOx obtained using background monitoring stations are probably more representative of population’s exposure than measurements of O_3_. In fact, O_3_ concentrations tend to vary within cities more than PM_2.5_, because of the scavenging of O_3_ by NO near roadways and principally, for its photochemical origin [37]. Thus, in the presence of a high density of local traffic, the measurement error is probably higher for exposure to O_3_ than for exposure to PM_2.5_. The effects of O_3_ could therefore be confounded by the presence of PM_2.5_ because of collinearity between the measurements of the two pollutants and the higher precision of measurements of PM_2.5_ [38].

Finally, a 0.7 % (95 % CI: 0.1–1.2 %) increase of ER admissions for every 10 grains/m^3^ increase of aeroallergens was observed at lag 1. This indicates a lower latency between the stimulus and the effect, compared to chemical pollutants. An apparent protective effect of aeroallergens at lag 4 shifted to the null when the models included meteorological adjustment variables. Adverse short-term effects of aeroallergens are supported by other studies [41, 42], although the time lags when excesses of ER admissions are observed vary according to a number of reasons, including differences in study populations, air pollutant mixtures, as well as exposure assessment and statistical methodologies applied.

A limitation in our study is that we used only one monitoring site to estimate air pollution concentrations. However, both chemical pollution and aeroallergen monitoring stations were located close to the children’s hospital. It is likely that children are referred to the closest hospital, especially in the case of acute health events that are captured by ER admissions, and we can therefore hypothesize that children lived at relative close distance to the monitoring area. Any measurement error in exposures due to spatial heterogeneity in airborne air pollution concentrations is more likely to bias risk estimates toward the null than in the opposite direction [43].

In conclusion, we observed consistent and positive associations of background NO_2_ and aeroallergen concentrations with ER admissions in children in a populated and heavily polluted city in western Italy. Our findings add to the existing evidence and call for urgent public health policies especially in the Po valley in northern Italy, one of the most polluted areas in Europe because of high emissions but also poor ventilation and precipitation especially in winter. Moreover, replacement of non-allergenic cultivated plant species and their management (for example frequent grassland mowing which limit the production of flowers and consequently of pollens) can reduce the concentrations of allergenic pollens in the air [44]. Air pollution reduction policies are also recommended in the protection and promotion of public health, especially in children.

## Abbreviations

COPD, chronic obstructive pulmonary disease; ER: emergency room: GAMs, generalized additive models; ICD, international classification of diseases; IQR: interquartile ranges, PACF partial autocorrelation function of the residuals; PM, particulate matter

## Additional file

Additional file 1: Table S1. Characteristics of the instruments of the meteorological station. **Figure S1.** Map of the city of Turin (grey line). The dots indicate the locations of the “Regina Margherita” Children’s Hospital (red), the aeroallergen sampling station (green), and the chemical air pollution monitoring and weather station (blue) (© 2015 Google maps). **Figure S2.** Estimates of the association of daily PM2,5 + aeroallergens (panel A), NO2 + aeroallergens (panel B), O3 + aeroallergens (panel C) with ER admissions for respiratory diseases at different time lags, adjusted for medium/long trend function, day of the week, influenza outbreaks, holidays and summer population decrease.* (DOCX 931 kb)
