# Supplementary material for: Air pollution, aeroallergens and admissions to pediatric emergency room for respiratory reasons in Turin, northwestern Italy
Source: BMC Public Health. 2016 Aug 5;16:722. doi: 10.1186/s12889-016-3376-3 (PMC4974813; doi:10.1186/s12889-016-3376-3)
Supplement: Additional file 1: Table S1. — Characteristics of the instruments of the meteorological station. Figure S1. Map of the city of Turin (grey line). The dots indicate the locations of the “Regina Margherita” Children’s Hospital (red), the aeroallergen sampling station (green), and the chemical air pollution monitoring and weather station (blue) (© 2015 Google maps). Figure S2. Estimates of the association of daily PM2,5 + aeroallergens (panel A), NO2 + aeroallergens (panel B), O3 + aeroallergens (panel C) with ER admissions for respiratory diseases at different time lags, adjusted for medium/long trend function, day of the week, influenza outbreaks, holidays and summer population decrease.* (DOCX 931 kb) [file 12889_2016_3376_MOESM1_ESM.docx]

SUPPLEMENTARY MATERIAL

Air pollution and admissions to pediatric emergency room for respiratory reasons in Turin, northwestern Italy

Roberto Bono^1^*****, Valeria Romanazzi^1^, Valeria Bellisario^1^, Roberta Tassinari^1^, Giulia Trucco^1^, Antonio Urbino^2^, Claudio Cassardo^3^, Consolata Siniscalco^4^, Pierpaolo Marchetti^5^, Alessandro Marcon^5^.

^1^ Department of Public Health and Pediatrics, University of Turin – via Santena, 5 bis, 10126, Turin, Italy.

^2^ Pediatrics Emergency, Regina Margherita Children’s Hospital - Piazza Polonia, 94, 10126, Turin, Italy.

^3^ Department of Physics. University of Turin - Via P. Giuria, 1, 10125 Turin, Italy.

^4^ Department of Life Sciences and Systems Biology - University of Turin - Viale P. A. Mattioli, 25, 10125 Turin, Italy.

^5^ Unit of Epidemiology and Medical Statistics, Department of Diagnostics and Public Health, University of Verona – Strada le Grazie, 8, Verona, Italy.

**Table S1:** Characteristics of the instruments of the meteorological station.

| **Meteorological instruments** | **Characteristics** |
| --- | --- |
| Psychrometer | model SIAP TM 7722, type with two platinum resistors Pt 100, measuring range -30/+50°C, accuracy 0.3°C |
| Hygrometer | model SIAP UM 5716, type hair hygrometer, range 0/100 %, accuracy ± 5% |
| Barometer | model SETRA 270, type capacity measurements, range 800/1100 hPa, accuracy ≤ ± 0.05% of scale |
| Microbarometer | model SETRA 239, type based on capacity, range -70/+70 Pa, accuracy ≤ ± 0.14% of scale |
| Pyranometer | model EPPLEY PSP, type thermopile on spectral band 0.285/2.800 μm, range 0/2800 W m^-2^, accuracy ±1% of scale |
| Pluviometer | model MTX PPI 030C, type tipping bucket, range 0/51 mm, accuracy ≤ ± 0.5% of scale |
| Anemometer | model MTX VVE 020C, type three-cup reel, range 0/50 m s^-1^, accuracy ≤ ± 1.5% of scale |
| Weathervane | model MTX VDI 030C, type gonioanemometer, range 0/360, accuracy ≤ ± 3° |
| Leaf wetness sensor | model MTX FAR751BA, type variable resistance, range 0/100 % |

**Figure S1.** Map of the city of Turin (grey line). The dots indicate the locations of the "Regina Margherita" Children’s Hospital (red), the aeroallergen sampling station (green), and the chemical air pollution monitoring and weather station (blue) (© 2015 Google maps).


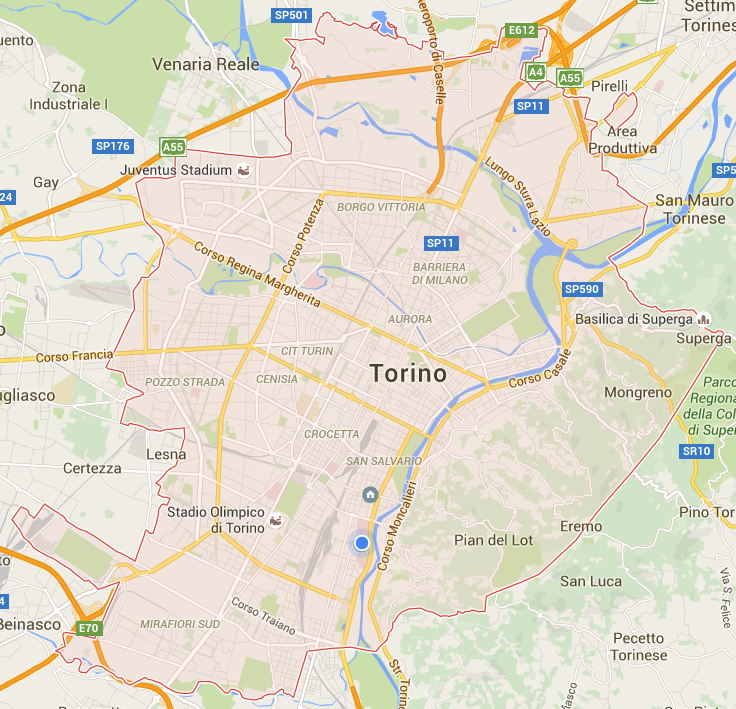


**Figure S2.** Estimates of the association of daily PM_2,5_ + aeroallergens (panel A), NO_2_ + aeroallergens (panel B), O_3_ + aeroallergens (panel C) with ER admissions for respiratory diseases at different time lags, adjusted for medium/long trend function, day of the week, influenza outbreaks, holidays and summer population decrease.*


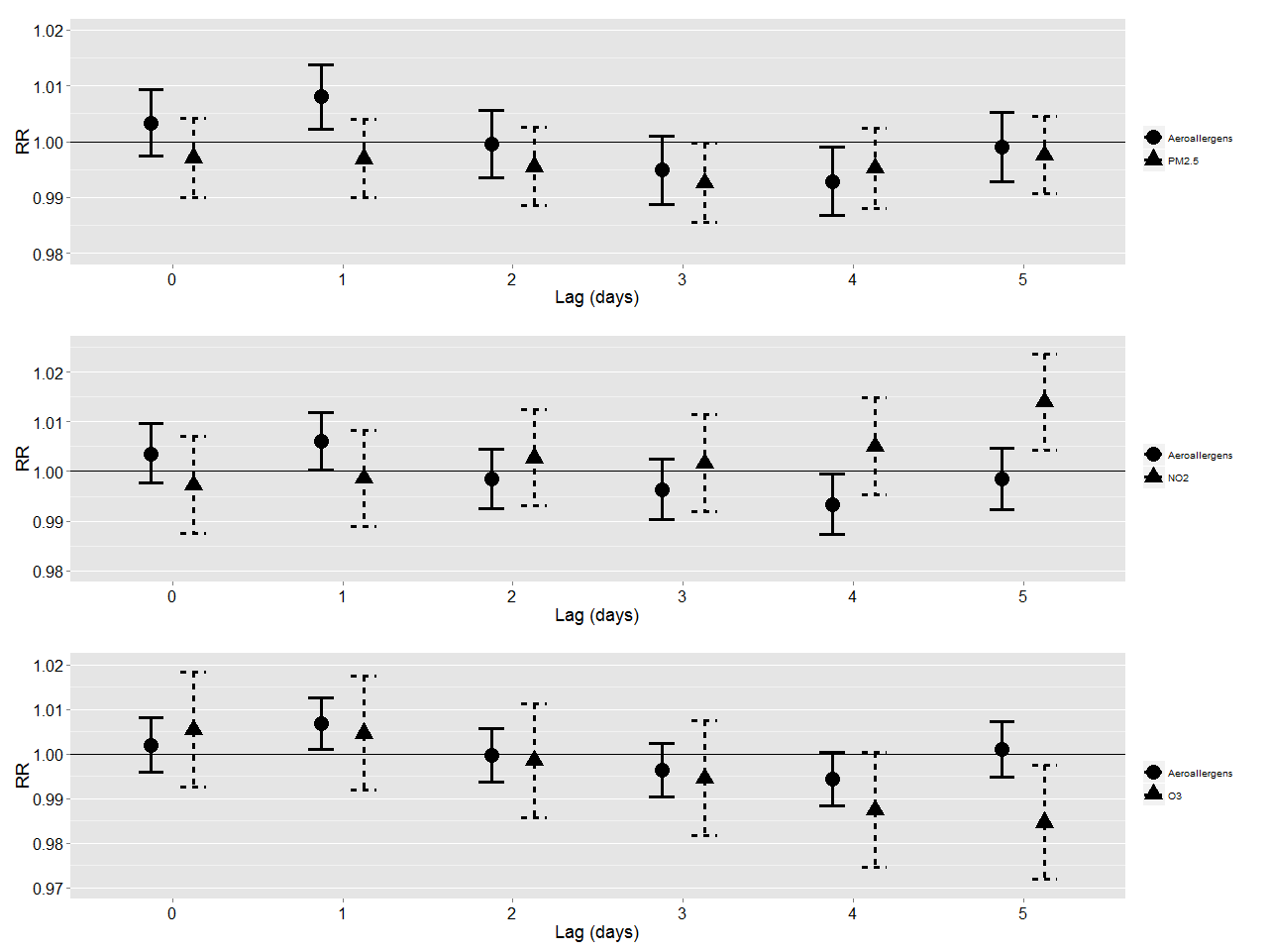


B

C

A

* *Two-pollutant models*, see “model C” in the Statistical analysis section of the article. Relative risks (RR) with 95%CIs are given for a 10 μg/m^3^ increase in PM_2,5_, NO_2_, O_3_ concentrations and a 10 grains/m^3^ increase in aeroallergen concentrations.
